# 2-(Carbazol-9-yl)acetic acid

**DOI:** 10.1107/S1600536809043463

**Published:** 2009-10-28

**Authors:** Min-Hao Xie, Pei Zou, Yong-Jun He, Ya-Ling Liu, Biao Huang

**Affiliations:** a Jiangsu Institute of Nuclear Medicine, Wuxi 214063, People’s Republic of China

## Abstract

In the title compound, C_14_H_11_NO_2_, the tricyclic aromatic ring system is essentially planar [maximum deviation = 0.025 (2) Å]. The dihedral angle between the two benzene rings is 2.8 (5)°, while the carboxyl group forms a dihedral angle of 88.5 (1)° with the pyrrole ring. Inter­molecular O—H⋯O hydrogen bonds may contribute to the overall stabilization of the crystal structure.

## Related literature

For the use of the title compound in high-performance liquid chromatography, see: Jinmao *et al.* (2001 ▶). For synthesis: Xie *et al.* (2006 ▶).
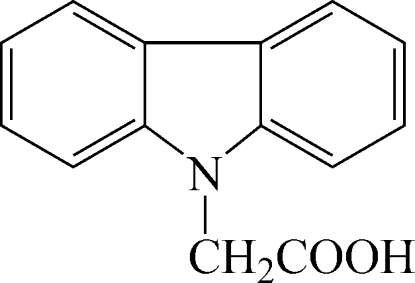


## Experimental

### 

#### Crystal data

C_14_H_11_NO_2_
*M* *_r_* = 225.24
Monoclinic, 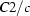

*a* = 32.067 (19) Å
*b* = 5.340 (3) Å
*c* = 13.134 (7) Å
β = 97.756 (8)°
*V* = 2229 (2) Å^3^
*Z* = 8
Mo *K*α radiation
μ = 0.09 mm^−1^
*T* = 93 K
0.40 × 0.30 × 0.08 mm

#### Data collection

Rigaku SPIDER diffractometer
Absorption correction: none
8360 measured reflections
2534 independent reflections
1749 reflections with *I* > 2σ(*I*)
*R*_int_ = 0.067

#### Refinement

*R*[*F* ^2^ > 2σ(*F* ^2^)] = 0.048
*wR*(*F* ^2^) = 0.093
*S* = 1.00
2534 reflections
159 parameters
H atoms treated by a mixture of independent and constrained refinement
Δρ_max_ = 0.22 e Å^−3^
Δρ_min_ = −0.25 e Å^−3^

### 

Data collection: *RAPID-AUTO* (Rigaku, 2004 ▶); cell refinement: *RAPID-AUTO*; data reduction: *RAPID-AUTO*; program(s) used to solve structure: *SHELXS97* (Sheldrick, 2008 ▶); program(s) used to refine structure: *SHELXL97* (Sheldrick, 2008 ▶); molecular graphics: *SHELXTL* (Sheldrick, 2008 ▶); software used to prepare material for publication: *SHELXTL*.

## Supplementary Material

Crystal structure: contains datablocks I, global. DOI: 10.1107/S1600536809043463/cs2123sup1.cif

Structure factors: contains datablocks I. DOI: 10.1107/S1600536809043463/cs2123Isup2.hkl

Additional supplementary materials:  crystallographic information; 3D view; checkCIF report

## Figures and Tables

**Table 1:** Hydrogen-bond geometry (Å, °)

*D*—H⋯*A*	*D*—H	H⋯*A*	*D*⋯*A*	*D*—H⋯*A*
O2—H2*O*⋯O1^i^	0.95 (3)	1.70 (3)	2.645 (2)	171 (2)

Symmetry code: (i) 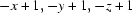.

**Fig. 1.**
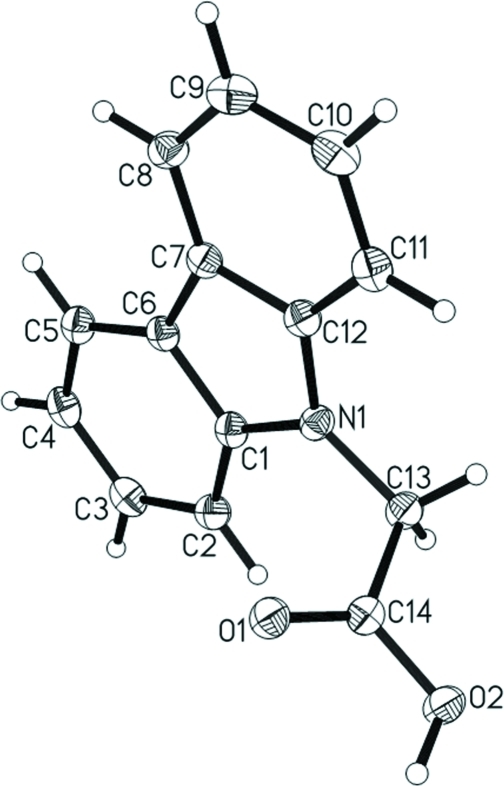
A view of the title compound with the atomic numbering scheme. Displacement ellipsoids were drawn at the 30% probability level.

## supplementary crystallographic information

### Comment

Carbazoles are ubiquitous structural subunits of numerous naturally occurring compounds as well as synthetic materials. The title molecule (Fig. 1), is useful as an important agent for determination of alcohols by high-performance liquid chromatography with fluorimetric detection after pre-column derivatization (Jinmao *et al.*, 2001; Xie *et al.*, 2006). The crystal structure shows that the tricyclic aromatic ring system is coplanar. The dihedral angle between the two benzene rings is 2.8 (5)°. The pyrrole ring makes dihedral angles of 1.5 (5)° and 1.3 (5)° with the benzene rings, respectively. The pyrrole ring and the carboxylic acid group (O1/C14/O2) are twisted to each other by a torsion angles of 88.5 (1)°. The crystal structure may be stabilized by intermolecular O2–H2O···O1_i_ [i= 1-x, 1-y, 1-z] hydrogen bonds.

### Experimental

The title compound was prepared by a method reported earlier (Xie *et al.*, 2006). The pure product (0.1 g) obtained was dissolved in 50% ethanol (10 ml). The solution was evaporated in air affording colourless platelet crystals suitable for X-ray analysis (yield: 67.2%).

### Refinement

Positional parameters of all the H atoms bonded to C atoms were calculated geometrically and were allowed to ride on the C atoms to which they are bonded,with C—H=0.95 and 0.99 Å for aromatic and methylene and with *U*_iso_(H) = 1.2*U*_eq_(aromatic,methylene) parent atoms. The carboxylic H atom was taken from a differnce density map and refined.

### Crystal data

**Table:**

C_14_H_11_NO_2_	*F*(000) = 944
*M**_r_* = 225.24	*D*_x_ = 1.343 Mg m^−^^3^
Monoclinic, *C*2/*c*	Mo *K*α radiation, λ = 0.71073 Å
Hall symbol: -C 2yc	Cell parameters from 2975 reflections
*a* = 32.067 (19) Å	θ = 3.1–27.5°
*b* = 5.340 (3) Å	µ = 0.09 mm^−^^1^
*c* = 13.134 (7) Å	*T* = 93 K
β = 97.756 (8)°	Platelet, colorless
*V* = 2229 (2) Å^3^	0.40 × 0.30 × 0.08 mm
*Z* = 8	

### Data collection

**Table:**

Rigaku SPIDER diffractometer	1749 reflections with *I* > 2σ(*I*)
Radiation source: Rotating Anode	*R*_int_ = 0.067
graphite	θ_max_ = 27.5°, θ_min_ = 3.1°
ω scans	*h* = −41→41
8360 measured reflections	*k* = −6→6
2534 independent reflections	*l* = −17→17

### Refinement

**Table:**

Refinement on *F*^2^	Secondary atom site location: difference Fourier map
Least-squares matrix: full	Hydrogen site location: inferred from neighbouring sites
*R*[*F*^2^ > 2σ(*F*^2^)] = 0.048	H atoms treated by a mixture of independent and constrained refinement
*wR*(*F*^2^) = 0.093	*w* = 1/[σ^2^(*F*_o_^2^) + (0.0136*P*)^2^ + 0.660*P*] where *P* = (*F*_o_^2^ + 2*F*_c_^2^)/3
*S* = 1.00	(Δ/σ)_max_ < 0.001
2534 reflections	Δρ_max_ = 0.22 e Å^−^^3^
159 parameters	Δρ_min_ = −0.25 e Å^−^^3^
0 restraints	Extinction correction: *SHELXL97* (Sheldrick, 2008), Fc^*^=kFc[1+0.001xFc^2^λ^3^/sin(2θ)]^-1/4^
Primary atom site location: structure-invariant direct methods	Extinction coefficient: 0.0006 (2)

### Fractional atomic coordinates and isotropic or equivalent isotropic displacement parameters (Å^2^)

**Table:**

	*x*	*y*	*z*	*U*_iso_*/*U*_eq_	
O1	0.45527 (4)	0.3887 (2)	0.43806 (10)	0.0305 (3)	
O2	0.48708 (4)	0.7517 (2)	0.40940 (10)	0.0322 (4)	
N1	0.38917 (5)	0.4955 (3)	0.28479 (11)	0.0248 (4)	
C1	0.39216 (6)	0.3113 (3)	0.21154 (13)	0.0240 (4)	
C2	0.42431 (6)	0.2678 (3)	0.15246 (14)	0.0285 (5)	
H2	0.4486	0.3715	0.1582	0.034*	
C3	0.41955 (6)	0.0674 (3)	0.08487 (14)	0.0309 (5)	
H3	0.4411	0.0330	0.0438	0.037*	
C4	0.38388 (6)	−0.0852 (4)	0.07555 (14)	0.0303 (5)	
H4	0.3814	−0.2204	0.0281	0.036*	
C5	0.35211 (6)	−0.0411 (3)	0.13498 (14)	0.0281 (5)	
H5	0.3279	−0.1456	0.1287	0.034*	
C6	0.35604 (6)	0.1578 (3)	0.20388 (13)	0.0235 (4)	
C7	0.33048 (6)	0.2527 (3)	0.27875 (13)	0.0245 (4)	
C8	0.29237 (6)	0.1775 (4)	0.30960 (14)	0.0291 (5)	
H8	0.2775	0.0374	0.2785	0.035*	
C9	0.27678 (6)	0.3113 (4)	0.38653 (15)	0.0338 (5)	
H9	0.2510	0.2615	0.4086	0.041*	
C10	0.29835 (6)	0.5181 (4)	0.43219 (15)	0.0344 (5)	
H10	0.2867	0.6077	0.4841	0.041*	
C11	0.33618 (6)	0.5962 (4)	0.40406 (14)	0.0303 (5)	
H11	0.3509	0.7360	0.4360	0.036*	
C12	0.35197 (6)	0.4611 (3)	0.32662 (14)	0.0255 (4)	
C13	0.42069 (6)	0.6818 (3)	0.31517 (14)	0.0277 (5)	
H13A	0.4072	0.8275	0.3440	0.033*	
H13B	0.4326	0.7395	0.2535	0.033*	
C14	0.45600 (6)	0.5897 (4)	0.39346 (14)	0.0257 (4)	
H2O	0.5081 (8)	0.687 (4)	0.4603 (19)	0.088 (9)*	

### Atomic displacement parameters (Å^2^)

**Table:**

	*U*^11^	*U*^22^	*U*^33^	*U*^12^	*U*^13^	*U*^23^
O1	0.0316 (8)	0.0297 (8)	0.0293 (8)	−0.0020 (6)	0.0000 (6)	0.0064 (6)
O2	0.0290 (8)	0.0318 (8)	0.0337 (8)	−0.0065 (7)	−0.0034 (6)	0.0074 (7)
N1	0.0252 (9)	0.0249 (9)	0.0238 (9)	−0.0024 (7)	0.0011 (7)	0.0003 (7)
C1	0.0286 (10)	0.0238 (11)	0.0187 (9)	0.0017 (8)	0.0001 (7)	0.0037 (8)
C2	0.0285 (11)	0.0308 (11)	0.0262 (10)	0.0003 (9)	0.0038 (8)	0.0068 (9)
C3	0.0373 (12)	0.0344 (12)	0.0214 (10)	0.0081 (9)	0.0057 (9)	0.0053 (9)
C4	0.0405 (12)	0.0283 (11)	0.0211 (10)	0.0068 (9)	0.0004 (9)	0.0011 (8)
C5	0.0330 (11)	0.0264 (11)	0.0235 (10)	0.0003 (9)	−0.0009 (8)	0.0019 (8)
C6	0.0262 (10)	0.0241 (11)	0.0190 (9)	0.0030 (8)	−0.0018 (7)	0.0031 (8)
C7	0.0252 (10)	0.0259 (10)	0.0210 (10)	0.0022 (8)	−0.0015 (7)	0.0040 (8)
C8	0.0261 (11)	0.0326 (12)	0.0274 (11)	−0.0014 (9)	−0.0004 (8)	0.0034 (9)
C9	0.0287 (11)	0.0460 (14)	0.0270 (11)	0.0019 (10)	0.0047 (8)	0.0056 (10)
C10	0.0354 (12)	0.0405 (13)	0.0275 (11)	0.0080 (10)	0.0043 (9)	−0.0007 (10)
C11	0.0356 (12)	0.0298 (11)	0.0240 (11)	0.0038 (9)	−0.0009 (8)	−0.0005 (9)
C12	0.0266 (10)	0.0269 (11)	0.0220 (10)	0.0015 (8)	−0.0001 (8)	0.0054 (8)
C13	0.0281 (10)	0.0275 (11)	0.0264 (10)	−0.0021 (8)	−0.0005 (8)	0.0033 (8)
C14	0.0282 (11)	0.0273 (11)	0.0221 (10)	−0.0015 (8)	0.0048 (8)	−0.0015 (8)

### Geometric parameters (Å, °)

**Table:**

O1—C14	1.224 (2)	C5—H5	0.9500
O2—C14	1.315 (2)	C6—C7	1.454 (2)
O2—H2O	0.95 (3)	C7—C8	1.397 (2)
N1—C1	1.388 (2)	C7—C12	1.411 (2)
N1—C12	1.391 (2)	C8—C9	1.385 (3)
N1—C13	1.436 (2)	C8—H8	0.9500
C1—C2	1.391 (2)	C9—C10	1.395 (3)
C1—C6	1.412 (2)	C9—H9	0.9500
C2—C3	1.386 (2)	C10—C11	1.379 (3)
C2—H2	0.9500	C10—H10	0.9500
C3—C4	1.397 (3)	C11—C12	1.397 (2)
C3—H3	0.9500	C11—H11	0.9500
C4—C5	1.385 (3)	C13—C14	1.506 (2)
C4—H4	0.9500	C13—H13A	0.9900
C5—C6	1.390 (2)	C13—H13B	0.9900
			
C14—O2—H2O	109.0 (14)	C9—C8—C7	118.62 (18)
C1—N1—C12	108.85 (15)	C9—C8—H8	120.7
C1—N1—C13	124.87 (16)	C7—C8—H8	120.7
C12—N1—C13	126.21 (16)	C8—C9—C10	121.03 (19)
N1—C1—C2	129.09 (17)	C8—C9—H9	119.5
N1—C1—C6	109.17 (16)	C10—C9—H9	119.5
C2—C1—C6	121.74 (17)	C11—C10—C9	121.81 (19)
C3—C2—C1	117.42 (18)	C11—C10—H10	119.1
C3—C2—H2	121.3	C9—C10—H10	119.1
C1—C2—H2	121.3	C10—C11—C12	117.21 (18)
C2—C3—C4	121.67 (18)	C10—C11—H11	121.4
C2—C3—H3	119.2	C12—C11—H11	121.4
C4—C3—H3	119.2	N1—C12—C11	129.33 (18)
C5—C4—C3	120.51 (18)	N1—C12—C7	108.75 (17)
C5—C4—H4	119.7	C11—C12—C7	121.91 (18)
C3—C4—H4	119.7	N1—C13—C14	113.56 (15)
C4—C5—C6	119.20 (18)	N1—C13—H13A	108.9
C4—C5—H5	120.4	C14—C13—H13A	108.9
C6—C5—H5	120.4	N1—C13—H13B	108.9
C5—C6—C1	119.46 (17)	C14—C13—H13B	108.9
C5—C6—C7	134.18 (18)	H13A—C13—H13B	107.7
C1—C6—C7	106.34 (16)	O1—C14—O2	124.31 (18)
C8—C7—C12	119.41 (18)	O1—C14—C13	123.49 (17)
C8—C7—C6	133.70 (18)	O2—C14—C13	112.19 (16)
C12—C7—C6	106.88 (16)		
			
C12—N1—C1—C2	−177.99 (18)	C12—C7—C8—C9	0.2 (3)
C13—N1—C1—C2	−0.9 (3)	C6—C7—C8—C9	178.84 (18)
C12—N1—C1—C6	1.23 (19)	C7—C8—C9—C10	0.4 (3)
C13—N1—C1—C6	178.30 (15)	C8—C9—C10—C11	−0.9 (3)
N1—C1—C2—C3	179.41 (16)	C9—C10—C11—C12	0.8 (3)
C6—C1—C2—C3	0.3 (3)	C1—N1—C12—C11	178.33 (18)
C1—C2—C3—C4	0.3 (3)	C13—N1—C12—C11	1.3 (3)
C2—C3—C4—C5	−0.6 (3)	C1—N1—C12—C7	−0.6 (2)
C3—C4—C5—C6	0.3 (3)	C13—N1—C12—C7	−177.59 (15)
C4—C5—C6—C1	0.3 (3)	C10—C11—C12—N1	−178.94 (17)
C4—C5—C6—C7	−177.68 (18)	C10—C11—C12—C7	−0.2 (3)
N1—C1—C6—C5	−179.87 (15)	C8—C7—C12—N1	178.71 (15)
C2—C1—C6—C5	−0.6 (3)	C6—C7—C12—N1	−0.3 (2)
N1—C1—C6—C7	−1.38 (19)	C8—C7—C12—C11	−0.3 (3)
C2—C1—C6—C7	177.91 (16)	C6—C7—C12—C11	−179.28 (16)
C5—C6—C7—C8	0.4 (4)	C1—N1—C13—C14	−82.2 (2)
C1—C6—C7—C8	−177.79 (19)	C12—N1—C13—C14	94.4 (2)
C5—C6—C7—C12	179.19 (19)	N1—C13—C14—O1	−9.8 (3)
C1—C6—C7—C12	1.01 (19)	N1—C13—C14—O2	171.34 (15)

### Hydrogen-bond geometry (Å, °)

**Table:**

*D*—H···*A*	*D*—H	H···*A*	*D*···*A*	*D*—H···*A*
O2—H2O···O1^i^	0.95 (3)	1.70 (3)	2.645 (2)	171 (2)

Symmetry codes: (i) −*x*+1, −*y*+1, −*z*+1.

## References

[bb1] Jinmao, Y., Bo, Zh. & Weibing, Zh. (2001). *J. Chromatogr. A*, **909**, 171–182.

[bb2] Rigaku (2004). *RAPID-AUTO* Rigaku Corporation, Tokyo, Japan.

[bb3] Sheldrick, G. M. (2008). *Acta Cryst.* A**64**, 112–122. 10.1107/S0108767307043930 18156677

[bb4] Xie, M. H., Qiu, A. Y., He, Y. J., Wu, J., Zhou, X. Q., Zou, P., Liu, Y. L. & Luo, S. N. (2006). *Chin. J. Anal. Chem.* **34**, S131–134.

